# Multiphysics simulations of a cylindrical waveguide optical switch using phase change materials on silicon

**DOI:** 10.1038/s41598-024-61473-w

**Published:** 2024-05-10

**Authors:** Alireza Malek Mohammad, Mahmoud Nikoufard, Senour Abdolghaderi

**Affiliations:** 1 Department of Electronics, Faculty of Electrical and Computer Engineering, University of Kashan, Kashan, 8731753153 Iran; 2 Nanoscience and Nanotechnology Research Center, University of Kashan, Kashan, 8731753153 Iran

**Keywords:** Optics and photonics, Applied optics, Optical materials and structures, Other photonics

## Abstract

This work presents the design and multiphysics simulation of a cylindrical waveguide-based optical switch using germanium-antimony-tellurium (GST) as an active phase change material. The innovative cylindrical architecture is theoretically analyzed and evaluated at 1550 nm wavelength for telecommunication applications. The dispersion relation is derived analytically for the first time to model the optical switch, while finite element method (FEM) and finite difference time domain (FDTD) techniques are utilized to simulate the optical modes, light propagation, and phase change dynamics. The fundamental TE_01_ and HE_11_ modes are studied in detail, enabling switching between low-loss amorphous and high-loss crystalline GST phases. Increasing the GST thickness is found to increase absorption loss in the crystalline state but also slows down phase transition kinetics, reducing switching speeds. A 10 nm GST layer results in competitive performance metrics of 0.79 dB insertion loss, 13.47 dB extinction ratio, 30 nJ average power consumption, and 3.5 Mb/s bit rate. The combined optical, thermal, and electrical simulation provides comprehensive insights towards developing integrated non-volatile photonic switches and modulators utilizing phase change materials.

## Introduction

Phase change materials (PCMs) based on germanium-antimony-tellurium (GST) alloys have emerged as promising candidates for photonic applications due to their unique properties. GST can reversibly and rapidly transition between amorphous and crystalline phases using electrical, optical, or thermal stimuli^1,2^. This phase transition is non-volatile in nature. GST has three stable phases—amorphous, distorted cubic and hexagonal. The hexagonal phase exhibits higher electrical conductivity but the cubic phase has more stable optical properties, making it preferred for practical applications^3–5^. The amorphous phase of GST has a disordered covalent bonding structure while the crystalline phase has more resonant bonding between atoms^6^. This difference in structure leads to large contrasts in optical properties like refractive index and absorption when transitioning between the phases^7^.

GST phase change materials have thus enabled several reconfigurable photonic devices including tunable color filters, absorbers, optical switches, and non-volatile photonic memories^8–12^. Integration with metamaterials and plasmonics has allowed dynamic control over terahertz resonances and optical antennas^6,13,14^. Harnessing the unique properties of GST and related PCMs holds great promise for integrated photonics, optical computing, and reconfigurable nanophotonics^15–17^. However, challenges remain in improving the switching time and integrating PCMs with CMOS-compatible nanofabrication processes. Further research and innovation in materials, device design, and manufacturing is needed to fully realize the potential of these fascinating phase change materials.

Nanoscale optical switches based on phase change materials like GST have promising applications in optical neural networks and quantum information processing. For example, Rios et al.^18^ demonstrated a single GST device capable of storing 8 bits using near-field optical switching. Integrating GST with plasmonic nanostructures has enabled dynamic control over optical properties. Wang et al.^19^ showed over four times electrical modulation of reflectance at 755 nm in an electrically switchable GST antenna using a silver plasmonic strip. Ahmadivand et al.^20^ controlled the optical response of a GST-loaded nanoparticle dimer, achieving large shifts in the resonance peak position and width. Various phase change photonic devices have been realized using GST. Wu et al.^21^ demonstrated a resonance-enhanced optical memory switch with 38 levels using sequential pulses. Wei et al.^22^ numerically verified a tunable GST mid-infrared absorber with 100% absorption. Wang et al.^23^ and Mahmoodi et al.^24^ simulated a low-loss, high-extinction ratio GST optical switch.

In this work, a cylindrical hybrid plasmonic optical switch using GST as the active phase change material is proposed and analyzed. The proposed structure, compatible with CMOS technology, supports the propagation of transverse electric (TE), transverse magnetic (TM), and hybrid electromagnetic modes. The field distributions of the fundamental HE_11_ and TE_01_ modes were determined by solving the Helmholtz equation, taking into account the material properties and geometrical parameters represented by the coefficients^25–29^. Subsequently, an optimization procedure was performed to calculate the optimal thickness of each layer, maximizing the overall performance of the device. Notably, for the first time, an analytical dispersion relation for the TE_01_ mode in the proposed switch structure was derived, providing a theoretical framework for understanding the mode propagation characteristics. The validity of this analytical model was further corroborated through full-wave simulations using the finite element method (FEM), ensuring the accuracy and reliability of the theoretical predictions. Multiphysics electrical and thermal modeling of the optimized geometry identified the voltage and timing parameters needed for reversible amorphous-crystalline phase switching of the GST. Key parameters including bit rate, energy consumption, extinction ratio, and insertion loss were extracted and compared favorably to prior GST-based photonic switches. This comprehensive multiphysics analysis and optimization of a cylindrical plasmonic GST switch provides an innovative architecture and detailed understanding of the thermal, electrical, and optical characteristics essential for future integrated non-volatile nanophotonics devices and circuits.

## Optical simulations

### Dispersion relation of circular GST-based optical switch

Phase change materials, GST alloys, have garnered significant attention for their unique optical properties and potential applications in reconfigurable photonic devices. These chalcogenide alloys exhibit a reversible phase transformation between the amorphous and crystalline states, accompanied by a substantial change in their refractive index and optical properties. This remarkable feature forms the basis for their utilization in phase change optical switches, enabling dynamic control and modulation of light propagation. The switching mechanism involves the application of electrical pulses to induce localized heating and subsequent phase transitions in the GST material. To switch from the amorphous to the crystalline phase (SET process), a relatively high current pulse is applied, heating the GST above its crystallization temperature. This thermal annealing allows the material to crystallize, resulting in a change in its refractive index and optical properties. Conversely, to switch from the crystalline to the amorphous phase (RESET process), a shorter but higher current pulse is employed. This pulse melts the GST material, and rapid quenching causes it to solidify into an amorphous state, exhibiting a different refractive index and optical characteristics. The refractive index contrast between the amorphous and crystalline phases of GST can be leveraged to control the propagation of light within the waveguide. By strategically positioning the GST material, optical properties such as phase and intensity can be modulated, enabling dynamic reconfiguration of photonic circuits and optical signal processing functionalities.

**Figure 1:**
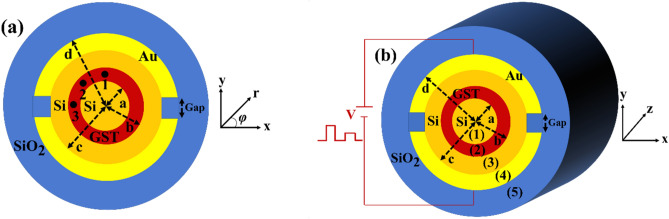
Cylindrical optical switch. (**a**) 2D cross-section of the proposed switch structure showing the GST active material surrounded by the silicon waveguide. (**b**) 3D view of the full optical switch device geometry.

The 2-dimensional (2D) and 3D schematic views of the proposed switch are shown in Fig. 1a,b, respectively. This cylindrical waveguide optical switch utilizes the phase change properties of GST and is designed for 1550 nm telecommunication wavelengths. As shown in Fig. 1, the switch has a cylindrical geometry with GST as the active material. When triggered, a phase transition in the GST shifts the waveguide between low loss and high loss states, thus performing optical switching. The unique cylindrical design allows for integration with fiber optics and takes advantage of the large refractive index change in GST upon switching.

The proposed optical switch consists of a cylindrical waveguide structure with four distinct layers, surrounded by SiO_2_ cladding, as shown in Fig. 1. The radii of the layers are labeled *a*, *b*, *c*, and *d* respectively. Layers 1 and 3 are composed of silicon (Si) which has a refractive index *n*_1_ = 3.477 + i0.0002 at the operating wavelength of 1.55 μm ^30–34^. Layer 2 is the phase change material GST which enables switching. In its amorphous phase, GST has a refractive index of *n*_2_ = 4.6 + i0.12, while in the crystalline phase it is *n*_2_ = 7.45 + i1.49^35–37^. The outermost layer 4 is gold (Au), patterned with two slits to form separate electrodes. Au has a refractive index of *n*_4_ = 0.58311 + i9.8639 at the target wavelength^38–40^. Finally, the cladding surrounding the waveguide structure is silicon dioxide (SiO_2_) which has a refractive index of *n*_5_ = 1.4657^41,42^. The refractive indices of Si, SiO_2_, and both amorphous and crystalline phases of GST were accurately modeled using the well-established Sellmeier's equation, which captures the dispersion behavior of dielectric materials. For the metallic Au layer, the complex refractive index was calculated employing the Drude model, suitable for describing the optical properties of metals. In the design of GST phase change optical switches, a crucial aspect is the implementation of two distinct gaps between the electrodes. These gaps serve a critical role in electrically isolating the electrodes. Simultaneously, the gap must be sufficiently narrow to facilitate efficient and localized heating of the GST material during the switching process.

To optically analyze the proposed cylindrical switch structure, we employ a cylindrical coordinate system. The wave propagation in each layer is described by the Helmholtz equation. For simplicity in the analysis, the thin slits in the outer gold layer are neglected, as they have a negligible effect on the effective refractive index (*n*_eff_) of the waveguide modes. With this approximation, the full cylindrical geometry can be analyzed using the Helmholtz equation to calculate *n*_eff_ and the optical properties of the propagating modes in the layered switch structure. The electric (**E**) and magnetic (**H**) fields can be determined from Helmholtz equations:

1 $$\left\{\begin{array}{c}{\nabla }^{2}{\bf{E}} + {(n{k}_{0})}^{2}\bf{E} = 0\\ {\nabla }^{2}{\bf{H}} + {(n{k}_{0})}^{2}\bf{H} = 0\end{array}\right.$$

where *k*_0_ = 2π/*λ* is the free space wave number, and *λ* is the free space wavelength, and *n* is the refractive index of each layer. In the cylindrical coordinate system, the electric (**E**) and magnetic (**H**) fields of the propagating optical waves can be expressed as:

2 $$\left\{\begin{array}{c}{\bf{E}} = {E}_{r}\left(r,\varphi ,z\right){\widehat{\mathbf{a}}}_{r} + {E}_{\varphi }\left(r,\varphi ,z\right){\widehat{\mathbf{a}}}_{\varphi } +{ E}_{z}\left(r,\varphi ,z\right){\widehat{\mathbf{a}}}_{z} \\ {\bf{H}} = {H}_{r}\left(r,\varphi ,z\right){\widehat{\mathbf{a}}}_{r} + {H}_{\varphi }\left(r,\varphi ,z\right){\widehat{\mathbf{a}}}_{\varphi } +{ H}_{z}\left(r,\varphi ,z\right){\widehat{\mathbf{a}}}_{z}\end{array}\right.$$

The electric and magnetic fields in longitudinal direction of all layers are written as follows;

3 $$\left\{\begin{array}{c}{E}_{zi} ={ E}_{zi}\left(r,\varphi \right) {e}^{-j\gamma z}\\ {H}_{zi} = { H}_{zi}\left(r,\varphi \right) {e}^{-j\gamma z}\end{array}\right. \,\,i=1,\dots ,5$$

The longitudinal field components (*E*_zi_ and *H*_zi_) in each layer of the cylindrical waveguide structure satisfy the Helmholtz eq.:

4 $$\left\{\begin{array}{c}{\nabla }^{2}{E}_{zi} +({k}_{i}^{2}-{\gamma }^{2}){E}_{zi}= 0\\ {\nabla }^{2}{H}_{zi} +({k}_{i}^{2}-{\gamma }^{2}){H}_{zi}= 0\end{array}\right.\,\, i, \dots , 5$$

where *k*_i_ = *k*_0_*n*_*i*_ is transverse wave number, *n*_*i*_ is the refractive index of each layer, and *γ* = *k*_0_(*n*_eff_ + j*n*_eff_) is the complex propagation constant. Taking advantage of the cylindrical geometry and applying the separation of variables technique, the Helmholtz Eq. (4) can be expanded as:

5 $$\left\{ {\begin{array}{*{20}c} {\frac{{d^{2} E_{zi} }}{{dr^{2} }} + \frac{{dE_{zi} }}{rdr} + \left( {n_{i}^{2} k_{0}^{2} - \gamma^{2} - \frac{{m^{2} }}{{r^{2} }}} \right)E_{zi} = 0} \\ {\frac{{d^{2} H_{zi} }}{{dr^{2} }} + \frac{{dH_{zi} }}{rdr} + \left( {n_{i}^{2} k_{0}^{2} - \gamma^{2} - \frac{{m^{2} }}{{r^{2} }}} \right)H_{zi} = 0} \\ {} \\ \end{array} } \right. \quad i = 1, \ldots , 5$$

The integer *m* represents the azimuthal mode number and describes the *φ*-dependence of the fields in the cylindrical waveguide. The solutions to the Helmholtz Eq. (5) for the azimuthal field components are Bessel functions (*J*(⋅) and *Y*(⋅)) in the dielectric layers (Si, and GST) and modified Bessel functions (*I*(⋅) and *K*(⋅)) in the Au layer.

6a $$\left\{\begin{array}{c}{E}_{z1} = \left[{A}_{11}{J}_{m}\left(\frac{{U}_{1}}{a}r\right)+{A}_{12}{Y}_{m}\left(\frac{{U}_{1}}{a}r\right)\right] {e}^{j\left(-\gamma z+m\varphi \right)}\\ { H}_{z1} = \left[{B}_{11}{J}_{m}\left(\frac{{U}_{1}}{a}r\right) {+ B}_{12}{Y}_{m}\left(\frac{{U}_{1}}{a}r\right)\right]{ e}^{j\left(-\gamma z+m\varphi \right) }\\ {U}_{1}^{2}= {a}^{2}\left({n}_{1}{k}_{0}^{2}-{\gamma }^{2}\right)\ge 0\end{array}\right. \quad 0\le r\le a$$

6b $$\left\{ {\begin{array}{*{20}c} {E_{z2} = \left[ {A_{21} J_{m} \left( {\frac{{U_{2} }}{b}r} \right) + A_{22} Y_{m} \left( {\frac{{U_{2} }}{b}r} \right)} \right] e^{{j\left( { - \gamma z + m\varphi } \right)}} } \\ {H_{z2} = \left[ {B_{21} J_{m} \left( {\frac{{U_{2} }}{b}r} \right) + B_{22} Y_{m} \left( {\frac{{U_{2} }}{b}r} \right)} \right] e^{{j\left( { - \gamma z + m\varphi } \right)}} } \\ {U_{2}^{2} = b^{2} \left( {n_{2} k_{0}^{2} - \gamma^{2} } \right) \ge 0} \\ \end{array} } \right. \quad a \le r \le b$$

6c $$\left\{ {\begin{array}{*{20}c} {E_{z3} = \left[ {A_{31} J_{m} \left( {\frac{{U_{3} }}{c}r} \right) + A_{32} Y_{m} \left( {\frac{{U_{3} }}{c}r} \right)} \right] e^{{j\left( { - \gamma z + m\varphi } \right)}} } \\ {H_{z3} = \left[ {B_{31} J_{m} \left( {\frac{{U_{3} }}{c}r} \right) + B_{32} Y_{m} \left( {\frac{{U_{3} }}{c}r} \right)} \right] e^{{j\left( { - \gamma z + m\varphi } \right) }} } \\ {U_{3}^{2} = c^{2} \left( {n_{3} k_{0}^{2} - \gamma^{2} } \right) \ge 0} \\ \end{array} } \right. \quad b \le r \le c$$

6d $$\left\{ {\begin{array}{*{20}c} {E_{z4} = \left[ {A_{41} I_{m} \left( {\frac{{U_{4} }}{d}r} \right) + A_{42} K_{m} \left( {\frac{{U_{4} }}{d}r} \right)} \right] e^{{j\left( { - \gamma z + m\varphi } \right) }} } \\ {H_{z4} = \left[ {B_{41} I_{m} \left( {\frac{{U_{4} }}{d}r} \right) + B_{42} K_{m} \left( {\frac{{U_{4} }}{d}r} \right)} \right] e^{{j\left( { - \gamma z + m\varphi } \right) }} } \\ {U_{4}^{2} = d^{2} \left( {n_{4} k_{0}^{2} - \gamma^{2} } \right) \ge 0} \\ \end{array} } \right. \quad c \le r \le d$$

6e $$\left\{\begin{array}{c}{E}_{z5} = \left[{A}_{51}{I}_{m}\left(\frac{{U}_{5}}{d}r\right) +{ A}_{52}{K}_{m}\left(\frac{{U}_{5}}{d}r\right)\right]{ e}^{j\left(-\gamma z+m\varphi \right)} \\ {H}_{z5} = \left[{B}_{51}{I}_{m}\left(\frac{{U}_{5}}{d}r\right) + {B}_{52}{K}_{m}\left(\frac{{U}_{5}}{d}r\right)\right] {e}^{j\left(-\gamma z+m\varphi \right)} \\ {U}_{5}^{2}= {d}^{2}\left({n}_{5}{k}_{0}^{2}-{\gamma }^{2}\right)\ge 0\end{array}\right. \quad d\le r$$

where *a*, *b*, *c*, and *d* are the radial boundaries of the cylindrical waveguide layers as labeled in Fig. 1, *U*_i_ represents the arguments of the Bessel and modified Bessel function solutions in each layer *i*, *A* and *B* are coefficients corresponding to the electric and magnetic field amplitudes, respectively.

The light propagation is primarily confined within layers 1–3, comprising the waveguide core regions. Consequently, Bessel functions, which are suitable for describing fields in cylindrical waveguides, are employed in Eqs. (6a–6c) to model the electric and magnetic field distributions in these layers accurately. However, in the Au and SiO_2_ layers (layers 4 and 5, respectively), the optical fields decay exponentially due to the metallic and dielectric nature of these materials. To account for this evanescent behavior, modified Bessel functions, which capture the exponential decay, are utilized in Eqs. (6d) and (6e) to describe the field distributions within these layers accurately. The optical field solutions can be simplified using the following boundary conditions: at *r* = 0, the fields must be finite, requiring *A*_12_ = *B*_12_ = 0, and as *r* → ∞, the fields decay to zero, requiring *A*_51_ = *B*_51_ = 0. To simplify the dispersion relation calculation, we set *m* = 0 and consider the TE_0μ_ mode. This azimuthally symmetric mode has no angular field dependence, providing physical insight while enabling straightforward analysis. Applying Maxwell's equations, the transverse electric and magnetic field components (*E*_ri_, *H*_φi_) in each layer can be determined from the corresponding longitudinal components (*E*_zi_, *H*_zi_) as:

7a $$\left\{ {\begin{array}{*{20}c} {E_{{\varphi 1}} = ~\frac{j}{{\left( {{\raise0.7ex\hbox{${U_{1} }$} \!\mathord{\left/ {\vphantom {{U_{1} } a}}\right.\kern-\nulldelimiterspace} \!\lower0.7ex\hbox{$a$}}} \right)}}\mu _{0} \omega \left[ {B_{{11}} J_{1} \left( {\frac{{U_{1} }}{a}r} \right)} \right]~e^{{ - j\gamma z}} } \\ {H_{{r1}} = ~\frac{j}{{\left( {{\raise0.7ex\hbox{${U_{1} }$} \!\mathord{\left/ {\vphantom {{U_{1} } a}}\right.\kern-\nulldelimiterspace} \!\lower0.7ex\hbox{$a$}}} \right)}}\gamma \left[ {B_{{11}} J_{1} \left( {\frac{{U_{1} }}{a}r} \right)} \right]~e^{{ - j\gamma z}} ~~~~~~} \\ \end{array} } \right. 0 \le r \le a$$

7b $$\left\{ {\begin{array}{*{20}c} {E_{{\varphi 2}} = ~\frac{j}{{\left( {{\raise0.7ex\hbox{${U_{2} }$} \!\mathord{\left/ {\vphantom {{U_{2} } b}}\right.\kern-\nulldelimiterspace} \!\lower0.7ex\hbox{$b$}}} \right)}}\mu _{0} \omega \left[ {B_{{21}} J_{1} \left( {\frac{{U_{2} }}{b}r} \right) + B_{{22}} Y_{1} \left( {\frac{{U_{2} }}{b}r} \right)} \right]~e^{{ - j\gamma z}} } \\ {H_{{r2}} = ~\frac{j}{{\left( {{\raise0.7ex\hbox{${U_{2} }$} \!\mathord{\left/ {\vphantom {{U_{2} } b}}\right.\kern-\nulldelimiterspace} \!\lower0.7ex\hbox{$b$}}} \right)}}\gamma \left[ {B_{{21}} J_{1} \left( {\frac{{U_{2} }}{b}r} \right) + B_{{22}} Y_{1} \left( {\frac{{U_{2} }}{b}r} \right)} \right]~e^{{ - j\gamma z~~~~~~~~}} } \\ \end{array} } \right. a \le r \le b$$

7c $$\left\{ {\begin{array}{*{20}c} {E_{\varphi 3} = \frac{j}{{\left( {{\raise0.7ex\hbox{${U_{3} }$} \!\mathord{\left/ {\vphantom {{U_{3} } c}}\right.\kern-0pt} \!\lower0.7ex\hbox{$c$}}} \right)}}\mu_{0} \omega \left[ {B_{31} J_{1} \left( {\frac{{U_{3} }}{c}r} \right) + B_{32} Y_{1} \left( {\frac{{U_{3} }}{c}r} \right)} \right] e^{ - j\gamma z} } \\ {H_{r3} = \frac{j}{{\left( {{\raise0.7ex\hbox{${U_{3} }$} \!\mathord{\left/ {\vphantom {{U_{3} } c}}\right.\kern-0pt} \!\lower0.7ex\hbox{$c$}}} \right)}}\gamma \left[ {B_{31} J_{1} \left( {\frac{{U_{3} }}{c}r} \right) + B_{32} Y_{1} \left( {\frac{{U_{3} }}{c}r} \right)} \right] e^{ - j\gamma z} } \\ \end{array} } \right. b \le r \le c$$

7d $$\left\{ {\begin{array}{*{20}c} {E_{\varphi 4} = \frac{j}{{\left( {{\raise0.7ex\hbox{${U_{4} }$} \!\mathord{\left/ {\vphantom {{U_{4} } d}}\right.\kern-0pt} \!\lower0.7ex\hbox{$d$}}} \right)}}\mu_{0} \omega \left[ {B_{41} I_{1} \left( {\frac{{U_{4} }}{d}r} \right) + B_{42} K_{1} \left( {\frac{{U_{4} }}{d}r} \right)} \right] e^{ - j\gamma z} } \\ {H_{r4} = \frac{j}{{\left( {{\raise0.7ex\hbox{${U_{4} }$} \!\mathord{\left/ {\vphantom {{U_{4} } d}}\right.\kern-0pt} \!\lower0.7ex\hbox{$d$}}} \right)}}\gamma \left[ {B_{41} I_{1} \left( {\frac{{U_{4} }}{d}r} \right) + B_{42} K_{1} \left( {\frac{{U_{4} }}{d}r} \right)} \right] e^{ - j\gamma z} } \\ \end{array} } \right. c \le r \le d$$

7e $$\left\{ {\begin{array}{*{20}c} {E_{\varphi 5} = \frac{j}{{\left( {{\raise0.7ex\hbox{${U_{5} }$} \!\mathord{\left/ {\vphantom {{U_{5} } d}}\right.\kern-0pt} \!\lower0.7ex\hbox{$d$}}} \right)}}\mu_{0} \omega \left[ {B_{51} K_{1} \left( {\frac{{U_{5} }}{d}r} \right)} \right] e^{ - j\gamma z} } \\ {H_{r5} = \frac{j}{{\left( {{\raise0.7ex\hbox{${U_{5} }$} \!\mathord{\left/ {\vphantom {{U_{5} } d}}\right.\kern-0pt} \!\lower0.7ex\hbox{$d$}}} \right)}}\gamma \left[ {B_{51} K_{1} \left( {\frac{{U_{5} }}{d}r} \right)} \right] e^{ - j\gamma z} } \\ \end{array} } \right. d \le r$$

To satisfy the boundary conditions at each interface (*r* = *a*, *r* = *b*, *r* = *c*, and *r* = *d*), the longitudinal magnetic field *H*_*z*_ must be continuous across the layer boundaries. By matching *H*_*z*_ at these interfaces, the number of unknown field coefficients can be reduced by half.

8a $${\text{If}} : r=a, { H}_{Z1}={H}_{Z2}\quad\text{then} \quad {B}_{11} = \frac{{B}_{21}{J}_{0}\left(\frac{{U}_{2}}{b}a\right)+ {B}_{22}{Y}_{0}\left(\frac{{U}_{2}}{b}a\right)}{{J}_{0}\left({U}_{1}\right)}$$

8b $$\text{If} : r = b, H_{Z2} = H_{Z3}\quad\text{then} \quad B_{31} = \frac{{B_{21} J_{0} \left( {U_{2} } \right) - B_{32} K_{0} \left( {\frac{{U_{3} }}{c}b} \right) + B_{22} Y_{0} \left( {U_{2} } \right)}}{{I_{0} \left( {\frac{{U_{3} }}{c}b} \right)}}$$

$${\text{If :}}\,{\text{r = c,}}\,{{H}}_{{{\text{z3}}}} {{ = H}}_{{{\text{z4}}}} \quad\text{then}$$

8c $${B}_{41} = \frac{{B}_{21}{F}_{11}+{B}_{22}{F}_{21}+{B}_{32}{F}_{31}+{B}_{42}{F}_{41}}{{J}_{0}\left(\frac{{U}_{3}}{c}b\right){I}_{0}\left(\frac{{U}_{4}}{d}c\right)}$$

8d $${F}_{11}={J}_{0}\left({U}_{2}\right){J}_{0}\left({U}_{3}\right), { F}_{21}={Y}_{0}\left({U}_{2}\right){J}_{0}\left({U}_{3}\right)$$

8e $${F}_{31}={J}_{0}\left(\frac{{U}_{3}}{c}b\right){Y}_{0}\left({U}_{3}\right)-{J}_{0}\left({U}_{3}\right){Y}_{0}\left(\frac{{U}_{3}}{c}b\right)$$

8f $${F}_{41}=-{J}_{0}\left(\frac{{U}_{3}}{c}b\right){K}_{0}\left(\frac{{U}_{4}}{d}c\right)$$

$${\text{If :}}\,{{r = d,}}\,{{H}}_{{{\text{z4}}}} {{ = H}}_{{{\text{z5}}}} \quad\text{then}$$

8g $${B}_{51} = \frac{{B}_{21}{F}_{12}+{B}_{22}{F}_{22}+{B}_{32}{F}_{32}+{B}_{42}{F}_{42}}{{J}_{0}\left(\frac{{U}_{3}}{c}b\right){I}_{0}\left(\frac{{U}_{4}}{d}c\right){K}_{0}\left(\frac{{U}_{5}}{d}c\right)}$$

8h $${F}_{12}={J}_{0}\left({U}_{2}\right){J}_{0}\left({U}_{3}\right){I}_{0}\left({U}_{4}\right), { F}_{22}={Y}_{0}\left({U}_{2}\right){J}_{0}\left({U}_{3}\right){I}_{0}\left({U}_{4}\right)$$

8i $${F}_{32}={J}_{0}\left(\frac{{U}_{3}}{c}b\right){Y}_{0}\left({U}_{3}\right){I}_{0}\left({U}_{4}\right)-{Y}_{0}\left(\frac{{U}_{3}}{c}b\right){J}_{0}\left({U}_{3}\right){I}_{0}\left({U}_{4}\right)$$

8j $${F}_{42}={J}_{0}\left(\frac{{U}_{3}}{c}b\right){I}_{0}\left(\frac{{U}_{4}}{d}c\right){K}_{0}\left({U}_{4}\right)-{J}_{0}\left(\frac{{U}_{3}}{c}b\right){I}_{0}\left({U}_{4}\right){K}_{0}\left(\frac{{U}_{4}}{d}c\right)$$

Similarly, applying boundary conditions requiring continuity of the transverse electric field *E*_φ_ across each interface allows determining the remaining unknown field coefficients. This can be achieved by solving the matrix equation:

9 $$\left[M\right]\left[C\right]=\left[0\right]\to \left[\begin{array}{cccc}{a}_{11}& {a}_{12}& 0& 0\\ {a}_{21}& {a}_{22}& {a}_{23}& 0\\ {a}_{31}& {a}_{32}& {a}_{33}& {a}_{34}\\ {a}_{41}& {a}_{42}& {a}_{43}& {a}_{44}\end{array}\right]\left[\begin{array}{c}{B}_{21}\\ {B}_{22}\\ {B}_{32}\\ {B}_{42}\end{array}\right]=\left[0\right]$$

Applying the boundary conditions and solving for the field coefficients, the dispersion relation for the cylindrical waveguide optical switch can finally be derived as:

$${\text{det}}\left(M\right)={a}_{11}{a}_{22}{a}_{33}{a}_{44}-{a}_{11}{a}_{22}{a}_{34}{a}_{43}-{a}_{11}{a}_{23}{a}_{32}{a}_{44}$$

10 $${+a}_{12}{a}_{21}{a}_{33}{a}_{44}+{a}_{12}{a}_{21}{a}_{34}{a}_{43}-{a}_{12}{a}_{23}{a}_{34}{a}_{41}+{a}_{11}{a}_{23}{a}_{34}{a}_{42}=0$$

The dispersion relation Eq. (10) was numerically solved to obtain the complex propagation constant *γ* for the supported modes. The first non-trivial solution corresponds to the fundamental HE_11_ mode. With *γ* determined, all the field coefficients can be calculated in terms of a single reference coefficient using Eqs. 8,9. The full 2D field profiles of *H*_z_, *E*_r_, and *E*_φ_ can then be evaluated across the cylindrical waveguide cross-section.

### The optimal thickness of the different layers

To optimize the inner Si layer thickness (layer 1), simulations were performed by sweeping this parameter from 100 to 1000 nm. The GST (layer 2), outer Si (layer 3), Au (layer 4) layers and gap thickness were fixed at 10 nm, 500 nm, 100 nm, and 1200 nm, respectively. The effective refractive index (*n*_eff_) was calculated for the HE_11_ and TE_01_ modes, which have the lowest cutoff frequencies. A minimum Au thickness of 20 nm is required considering the electromagnetic field penetration depth, while a thickness of 100 nm is chosen to facilitate efficient heat transfer during the switching process involving the phase transition of the GST layer^43,44^. The 1200 nm gap thickness has a negligible effect on *n*_eff_ but enables heat confinement for phase change^43^.

**Figure 2:**
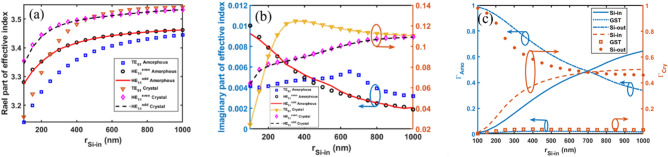
Effective refractive index of the proposed optical switch. (**a**) Real and (**b**) imaginary parts are shown for the fundamental HE_11_ and TE_01_ modes in both amorphous and crystalline phases of the GST material (**c**) confinement factor (*Γ*) of different layers for both amorphous and crystalline phases of the GST material of TE_01_ mode.

Figure 2a,b shows the simulated Re(*n*_eff_) and Im(*n*_eff_) parts of the effective refractive index and confinement factor (*Γ*) as a function of the inner Si layer thickness (0 ≤ *r* ≤ *a*) for both amorphous and crystalline phases of the GST material. As shown in Fig. 2a, Re(*n*_eff_) increases for both HE_11_ and TE_01_ modes as the inner Si layer (layer 1) thickness grows from 100–500 nm for both amorphous and crystalline GST phases. In this thickness range, optical power transfers to the inner Si layer, which can be confirmed by Fig. 2c. Above 500 nm, Re(*n*_eff_) saturates as the optical power becomes confined in the inner Si. The refractive index of crystalline GST is higher than amorphous GST, so Re(*n*_eff_) is greater in the crystalline phase. As the inner Si layer gets thicker, Re(*n*_eff_) approaches the Si index since more electromagnetic fields become concentrated in layer 1. Figure 2b shows Im(*n*_eff_) for the TE_01_ and HE_11_ modes. The trends depend on how the optical power becomes distributed between the GST and inner Si layers. For the HE_11_ mode, Im(*n*_eff_) decreases with increasing inner Si thickness in the amorphous GST phase, while it increases for crystalline GST. However, Im(*n*_eff_) behaves differently for the TE_01_ mode—it initially increases then decreases with increasing inner Si thickness for both GST phases. This distinct behavior arises from differences in how the optical field becomes concentrated within the GST layer for each mode. Figure 2c depicts the confinement factors of different layers as a function of the inner Si layer thickness for both amorphous and crystalline phases of the GST material, considering the TE_01_ mode. As the inner Si layer thickness increases, a larger fraction of the optical power is confined within this layer, while the confinement factor in the GST layer remains relatively constant. Consequently, the optical power confinement in the outer Si layer decreases, correspondingly.

**Figure 3:**
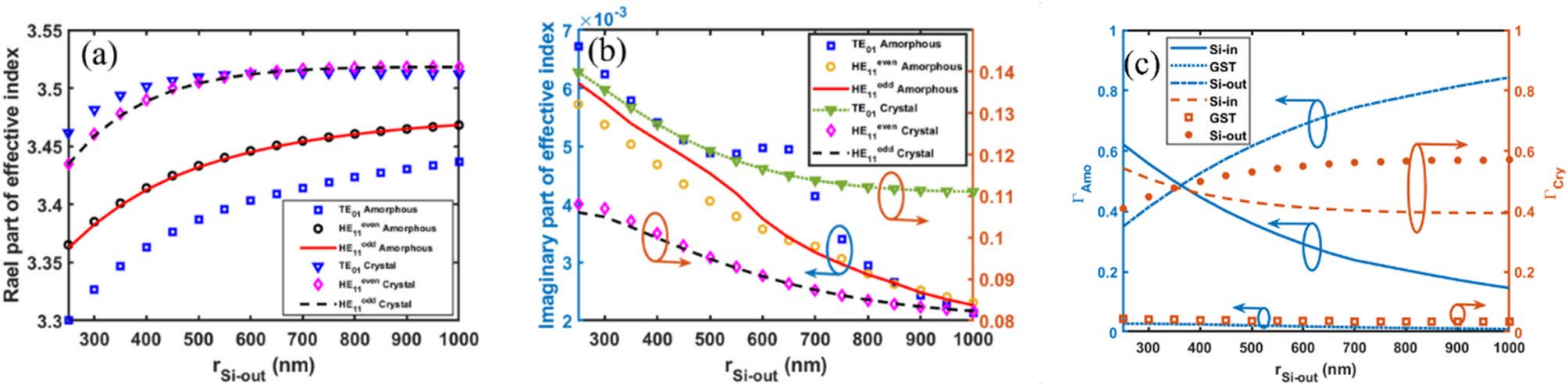
Effective refractive index of the proposed optical switch as a function of outer Si layer thickness. (**a**) Real and (**b**) imaginary parts are shown for the HE_11_ and TE_01_ modes in both amorphous and crystalline phases of the GST (**c**) confinement factor (*Γ*) of different layers for both amorphous and crystalline phases of the GST material of TE_01_ mode.

To optimize the outer Si layer thickness (*b* < *r* < *c*), simulations were performed sweeping this parameter from 250 to 1000 nm. The other layers were fixed with the following thicknesses: GST at 10 nm (layer 2), inner Si (layer 1) at 500 nm, Au (layer 4) at 100 nm, and gap at 1200 nm. Figure 3a,b shows the simulated Re(*n*_eff_) and Im(*n*_eff_) as a function of outer Si thickness for the TE_01_ and HE_11_ modes. In the amorphous GST phase, Re(*n*_eff_) increases smoothly with outer Si (layer 3) thickness for both modes. For crystalline GST, Re(*n*_eff_) rises until 500 nm thickness of outer Si, then saturates. Because the optical power concentrates in the outer Si layer for both phases, as shown in Fig. 3c.Guided modes only exist above 250 nm outer Si thickness and below this value, the TE_01_ and HE_11_ modes cannot be supported. The imaginary part of the effective refractive index (Im(*n*_eff_)) decreases as the outer Si thickness increases and then saturates for both phases of GST and for both TE_01_ and HE_11_ modes. This saturation behavior suggests that beyond a certain thickness, increasing the outer Si layer does not significantly alter the optical field distribution within the waveguide structure. Figure 3c illustrates the confinement factors of different layers as a function of the outer Si layer thickness for both amorphous and crystalline phases of the GST material, considering the TE_01_ mode. As the outer Si thickness increases, the optical power confinement in the outer Si layer increases, while the confinement factors in the GST and inner Si layers decrease, correspondingly.

Based on the trends in Fig. 2,3, the inner and outer Si layer thicknesses are set at 500 nm and 1000 nm, respectively, where the Re(*n*_eff_) is saturated. This provides optimal performance for amorphous and crystalline GST phases.

**Figure 4:**
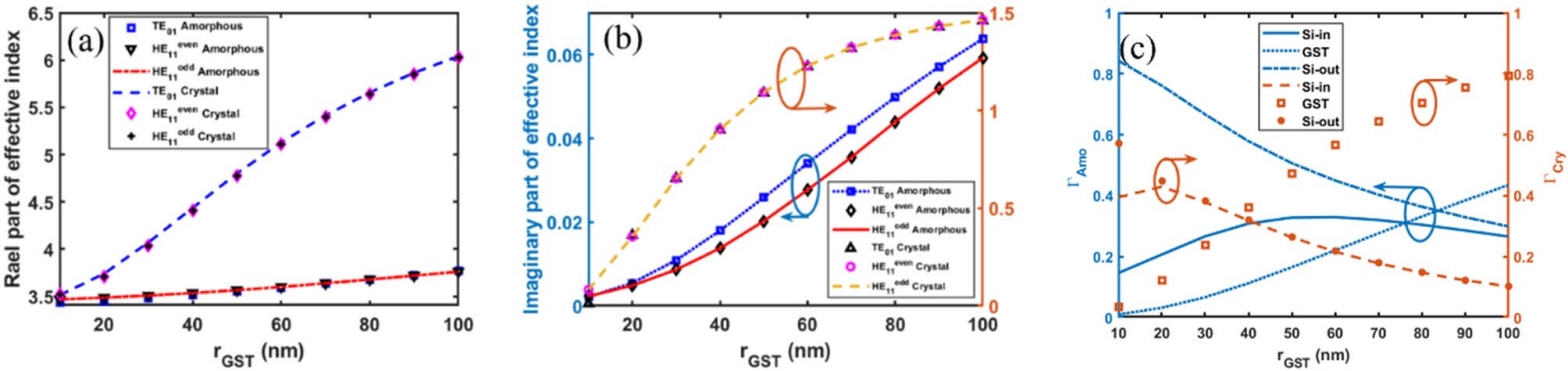
Effective refractive index of the proposed optical switch as a function of GST layer thickness. (**a**) Real and (**b**) imaginary parts are shown for the HE_11_ and TE_01_ modes in both amorphous and crystalline GST phases (**c**) confinement factor (*Γ*) of different layers for both amorphous and crystalline phases of the GST material of TE_01_ mode.

To determine the optimal GST layer thickness, simulations were performed sweeping this parameter from 10 to 100 nm. The Au metal layer and gap dimensions were fixed at 100 nm, and 1200 nm, respectively. Figure 4 shows Re(*n*_eff_) and Im(*n*_eff_) as a function of the GST layer thickness. As depicted in Fig. 4a, for the crystalline phase of GST, Re(*n*_eff_) increases toward the refractive index of the GST layer with increasing GST thickness for both HE_11_ and TE_01_ modes. This behavior is attributed to enhanced optical confinement within the GST layer. Conversely, in the amorphous phase, Re(*n*_eff_) exhibits minimal dependence on GST thickness due to the relatively small increase in optical power confinement within the GST layer. Figure 4b shows Im(*n*_eff_) rises with increasing GST thickness for both phases and modes, indicating higher optical loss. The loss is much greater in crystalline versus amorphous GST for example about 46 times higher at 50 nm GST thickness. This enables switching between crystalline and amorphous phases. Figure 4c depicts the confinement factor of different layers as a function of the GST layer thickness. As the GST layer thickness increases, the confinement factor within the GST layer rises, while the optical power confinement in the outer Si layer decreases. Maximal power confinement in the inner Si layer occurs at GST thicknesses of 20 nm and 60 nm for the crystalline and amorphous phases, respectively. However, a trade-off between switching speed and GST layer thickness led to the selection of a 10 nm thickness for the GST layer.

To determine the optimal Au layer thickness (*c* < *r* < *d*), simulations were performed sweeping this parameter from 10 to 100 nm. The gap distance was fixed at 1200 nm. The simulation results varying the Au layer thickness has minimal effect on Re(*n*_eff_) and Im(*n*_eff_) for both GST phases and modes, as shown in supplementary. This indicates negligible optical power propagates in the Au layer, which is due to the optimization of the inner and outer Si layer thicknesses. Based on these results and thermal simulations in the next section, the Au thickness is set to 100 nm. This enables voltage application without melting the electrodes^45,46^.

To determine the optimal gap thickness, simulations were performed sweeping this dimension from 100 to 1000 nm. The results, as shown in the supplementary Fig. s2, indicate that increasing the gap thickness has a negligible effect on the Re(*n*_eff_) and Im(*n*_eff_) for both amorphous and crystalline phases of the GST material. This suggests that the gap does not substantially alter the optical modes within the simulated range. However, the main role of the gap is to divide the Au layer, enabling the formation of electrodes for applying the voltage required for electrical switching. Since both Au and crystalline GST absorb optical power, the gap thickness is selected as 1200 nm. This value minimizes absorption losses while providing sufficient space for the electrode integration. The chosen gap dimension of 1200 nm facilitates efficient electrical switching by allowing localized heat generation within the GST layer, without perturbing the optical properties of the waveguide modes. This optimal design ensures reliable phase transition and switching capabilities while maintaining the desired optical performance.

### Longitudinal and lateral optical simulations of the cylindrical switch

**Figure 5:**
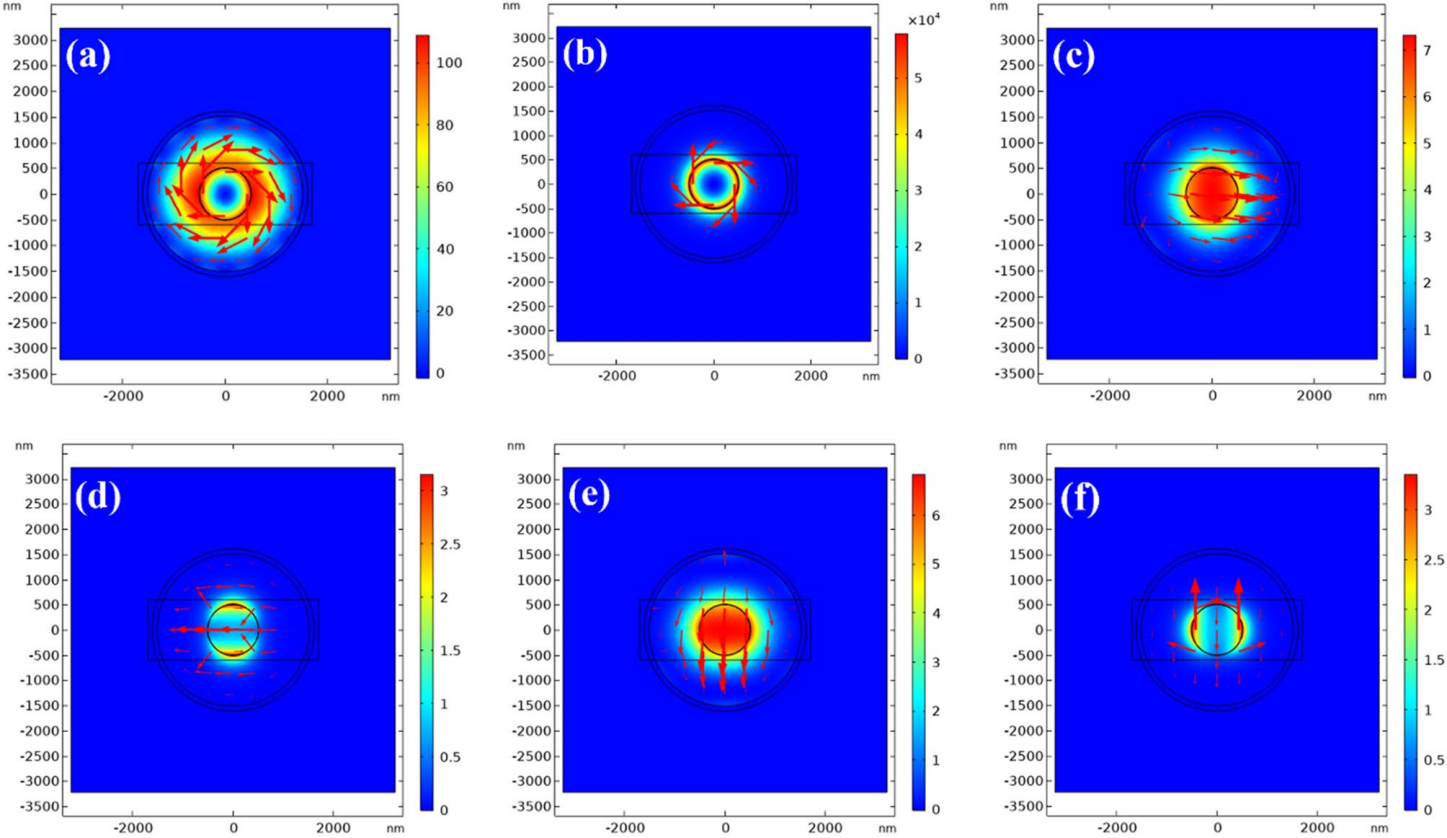
Optical power distribution for the TE_01_ and HE_11_ modes. TE_01_ mode in the (**a**) amorphous and (**b**) crystalline GST phases. Even HE_11_ mode pattern in the (**c**) amorphous and (**d**) crystalline phases. Odd HE_11_ mode pattern in the (**e**) amorphous and (**f**) crystalline phases. Peaks of optical power in *x*- or *y*-direction define the even and odd pattern. Arrows show the electrical field directions.

To examine the optical power distribution of the TE_01_ (even and odd) and HE_11_ modes in both amorphous and crystalline GST phases, simulations were performed as shown in Fig. 5. The previously optimized layer thicknesses were used to calculate the field patterns for each mode. Comparing the amorphous and crystalline results provides insight into how the phase change affects modal confinement. Figure 5 shows the simulated optical power distribution and electric field directions across the 2D switch structure. Figure 5a,b show the TE_01_ mode in amorphous and crystalline GST, respectively. In the crystalline phase, optical power is strongly confined in the GST layer, while in the amorphous phase it is distributed across both the GST and outer Si (layer 3). The imaginary part of the effective index is 46 times higher in crystalline versus amorphous GST. For the even and odd HE_11_ modes, optical power is uniformly distributed between the inner Si (layer 1) and GST layers for amorphous GST (Fig. 5c,e), while it localizes in the GST for crystalline (Fig. 5d,f). The effective indices for all modes and phases are listed in Table 1. The localization of optical power within the GST and/or Si layers is discussed in the previous subsection, where the layer thicknesses were varied and their impact on the effective refractive index was analyzed.

**Table 1:** Effective refractive index at 1.55 μm wavelength for the TE_01_, even HE_11_, and odd HE_11_ modes in the optimized cylindrical waveguide switch design. Results are shown for both amorphous and crystalline phases of the GST layer.

*N*_eff_	Amorphous	Crystalline
TE_01_	3.4369 + i0.0024126	3.5121 + i0.11104
HE_11_ (even)	3.4683 + i0.0022628	3.5181 + i0.082271
HE_11_ (odd)	3.4682 + i0.0023114	3.5181 + i0.082307

**Figure 6:**
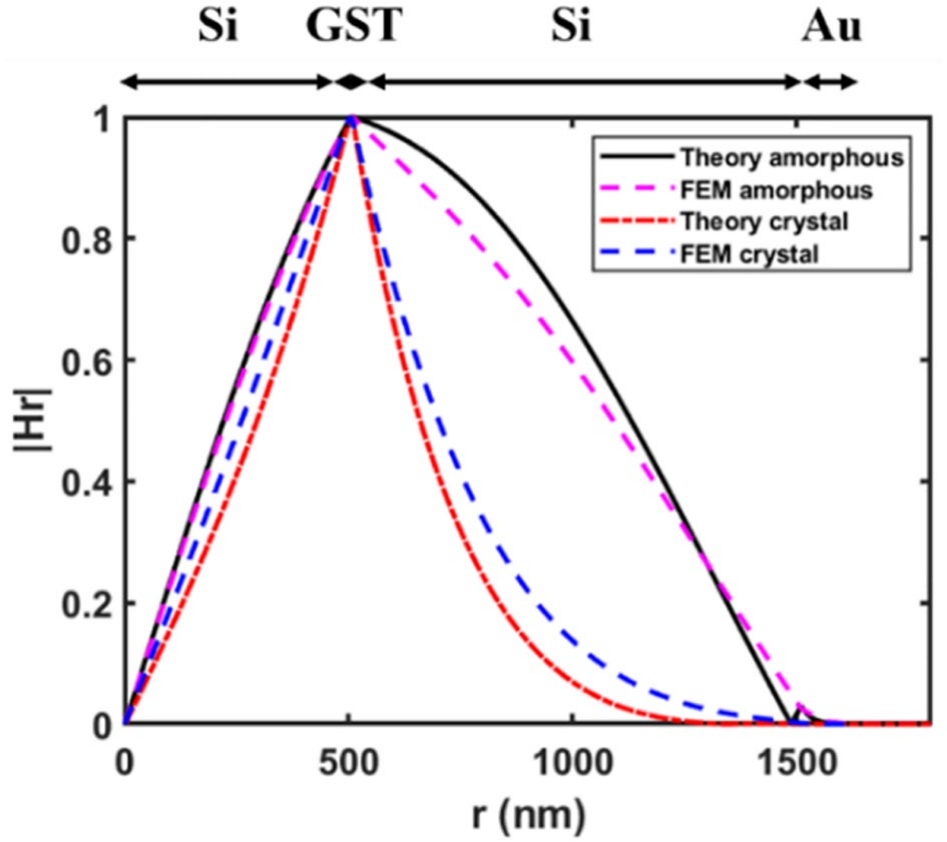
Radial magnetic field profile *H*_r_ for the TE_01_ mode in amorphous and crystalline GST phases. The profiles are determined analytically using Eqs. (1–10) and numerically using FEM.

Figure 6 shows the magnetic field profile along the radial direction for the TE_01_ mode, calculated both numerically using FEM and analytically using Eqs. (1–10). For the analytical approach, the propagation constant γ was derived by solving the equations. This γ was substituted back into the field expressions and plotted in MATLAB software. Comparing the two methods, helps verify the accuracy of the proposed switch model and optimized design parameters. In the amorphous GST phase, the radial magnetic field (*H*_r_) continuously increases from *r* = 0 to 510 nm, corresponding to the inner Si layer (layer 1). It then decreases in the GST layer (layer 2), reaching zero in the Au metal region (layer 4) between 1500–1600 nm. The crystalline GST phase follows a similar trend, with the key difference that the field steeply drops to zero within the GST layer itself. The numerically simulated *H*_r_ reaches zero at 1467 nm radius, while the analytically derived field extends slightly further to 1550 nm radius before becoming zero. As shown in Table 2, the average difference between the theoretical and numerically simulated results is about 0.04% for the Re(*n*_eff_) and 0.3% for the Im(*n*_eff_). Based on Table 2 and Fig. 6, the analytical model shows close agreement with the finite element method (FEM) simulations, with only a small difference between the two approaches.

**Table 2:** Comparison of *N*_eff_ and *γ* for the TE_01_ mode in the optimized optical switch design. Results are shown at 1.55 μm wavelength from both the analytical model (theory) and numerical FEM simulations.

	Amorphous	Crystalline
*N*_eff_ (Theory)	3.43392 + i0.00199	3.51236 + i0.11116
*N*_eff_ (FEM)	3.43630 + i0.00198	3.51220 + i0.11105
(Theory)	13.91997e + 3 + i8.06680e + 3	14.23794e + 6 + i450.60573e + 3
γ (FEM)	13.92962e + 3 + i8.02626e + 3	14.23729e + 6 + i450.15982e + 3

**Figure 7:**
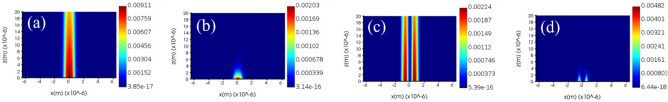
Optical power distribution along the length for the HE_11_ even mode **(a, b)** and TE_01_ (**c**, **d**) mode. Results are shown in the (**a**, **c**) amorphous and (**b**, **d**) crystalline GST phases. The optical power propagation in the cylindrical switch is analyzed to determine the required device length.

**Figure 8:**
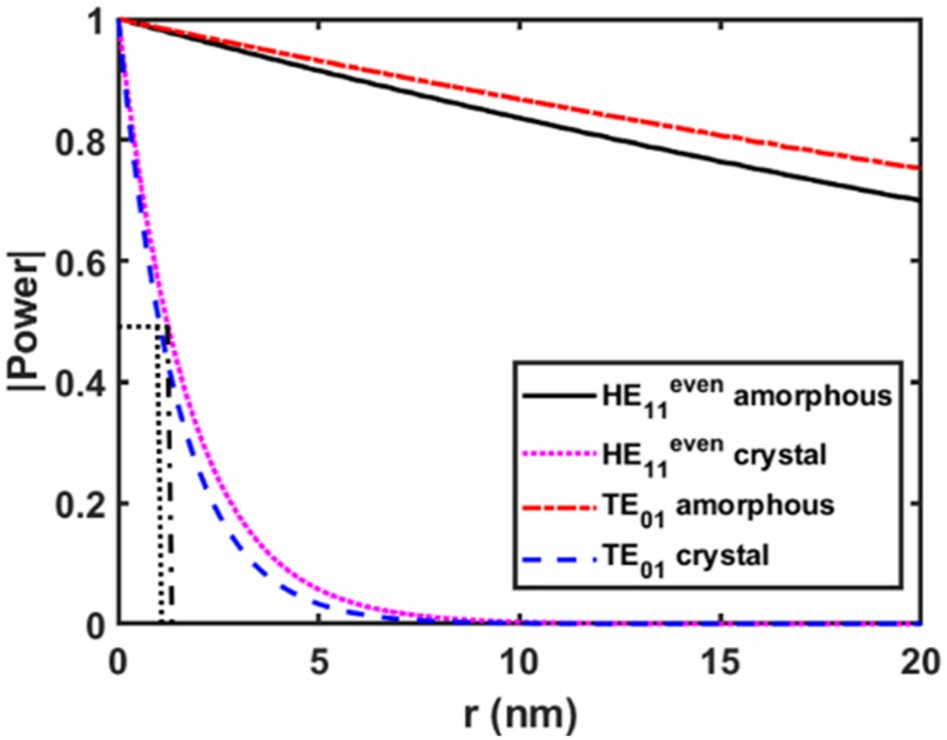
Simulated optical power distribution in the optimized switch design for the even HE_11_ and TE_01_ modes. Results are shown for the amorphous and crystalline GST phases. The power is plotted at the midpoint of the inner Si and outer Si layers, respectively.

To determine the appropriate optical switch length, light propagation simulations were performed using the finite-difference time-domain (FDTD) method. Figure 7 shows the simulated optical power along the *xz*-plane for the even HE_11_ and TE_01_ modes in both amorphous and crystalline GST phases. Analyzing the power distribution along the length helps identify the minimum length needed for efficient optical switching. The comparison between amorphous and crystalline phases also reveals differences in optical confinement that impact device length. In the amorphous GST phase, Fig. 7a,c show light propagates through the switch with minimal loss. However, in the crystalline phase (Fig. 7b,d), significant loss occurs, with the power fully decayed after a few micrometer. Figure 7a indicates the HE_11_ mode power is primarily confined in the inner Si layer (layer 1). In contrast, Fig. 7c shows the TE_01_ mode propagates around the GST layer (layer 2). Figure 8 shows the simulated optical power propagation along the switch length for the HE_11_ and TE_01_ modes in the GST crystalline and amorphous phases at the midpoint of the inner Si and outer Si layers, respectively. In the amorphous phase, the optical power drops about 30% for both modes within a 20 μm length. However, in the crystalline phase the optical power rapidly decays within 10 μm. Specifically, the optical power reduces by over 50% within just 1.2 μm and 1.05 μm for the HE_11_ and TE_01_ modes, respectively in crystalline GST. In contrast, the amorphous phase, optical power only drops around 5% over the same distance. Based on these results, switch lengths of 1.2 μm and 1.05 μm are chosen for the HE_11_ and TE_01_ modes respectively to enable efficient switching between crystalline and amorphous phases.

### Electrical and thermal simulations

Phase change of GST can be induced by raising its temperature for a set time before cooling. This is typically achieved via optical or electrical stimulation^47^. In this work, electrical stimulation is utilized for switching. Voltage applied to the gold layers causes current to flow through the GST, heating it up. The elevated temperature triggers the reversible transition between amorphous and crystalline phases. This switching allows the proposed cylindrical waveguide structure to selectively transmit or reflect certain wavelengths by altering the GST refractive index.

To simulate the electrical heating process, the heat transfer equation (Eq. 11) was coupled to the electric current equation (Eq. 12) using FEM^48^.

11a $$\rho {C}_{{\text{p}}}\frac{{dT}}{{dt}}+\rho {C}_{{\text{p}}}{u}_{{\text{trans}}}\cdot\nabla T+\nabla \cdot\left(q+{q}_{{\text{r}}}\right)=-\mathrm{\alpha }T\frac{dS}{dt}+{\text{Q}}$$

11b $$Q=-\alpha T\frac{dS}{dt}$$

where *ρ* is the density (kg/m^3^), *C*_p_ is the heat capacity (J/kg.K), *k* is the thermal conductivity (W/m⋅K), *T* is the absolute temperature (K), *u*_trans_ is the translational velocity (m/s), *q* is the heat flux by conduction (W/m^2^), *q*_r_ is the heat flux by radiation (W/m^2^), *α* is the coefficient of thermal expansion (1/K), *S* is second Piola–Kirchhoff stress tensor (Pa), and *Q* is the additional heat sources (W/m^3^). For steady-state conditions, the temperature is constant over time. Therefore, the transient heat transfer Eq. (11a) reduces to the steady-state form Eq. (11b).

To simulate joule heating in the phase change material, the electric current and heat transfer physics were coupled. The Poisson’s equation [Eq. (12)] was used to calculate current distribution by the applied voltage. The heat transfer equation [Eq. (11)] then modeled thermal conduction and temperature distribution in the switch structure.

12 $$\nabla \cdot \left(\sigma \nabla V\right)=0$$

where *σ* is the electrical conductivity and *V* is the electric potential. By iteratively solving thermal-electrical model, the temperature profile arising from the applied voltage was determined. This enables predicting the amount of voltage requirements for phase change. As temperature variations induce phase change in the GST layer, the temperature-dependent properties of the Si, GST, and Au layers were entered into the concerned equations. This ensures accuracy when simulating across the wide temperature range required for amorphous to crystalline (or vice versa) switching. The electrical and thermal parameters of different layers are presented in supplementary.

Yu et al.^49^ investigated the relationship between GST thickness and required switching time. Their findings indicate that for a 10 nm thick GST layer, the crystallization threshold temperature is 413 K, while the amorphization threshold temperature is 819 K. Thicker GST layers need longer dwell times above the crystallization temperature for phase change, decreasing the switching speed. Wang et al.^50^ also studied the transient temperatures needed for amorphous-to-crystalline and crystalline-to-amorphous switching. Picosecond pulses can melt and quench crystalline GST into the amorphous phase since the atomic bonds do not have time to rearrange. However, nanosecond pulses are required for crystallization to allow resonant bonding. Meanwhile, experimental data were used for the temperature-dependent heat capacity, thermal conductivity, and electrical conductivity of gold layer, presented in supplementary^51,52^.

Applying tailored electrical pulses allows temporally controlling the phase change in GST between amorphous and crystalline states. The pulse parameters for amorphous-to-crystalline switching are: source voltage *V*_src_ = 5V, offset voltage *V*_off_ = 0V, delay time *t*_d_ = 0 ns, rise time *t*_r_ = 1 ns, fall time *t*_f_ = 1 ns, pulse width *p*_w_ = 140 ns, and period *T*_per_ = 300 ns. For crystalline-to-amorphous transition, the parameters are: *V*_src_ = 15V, *t*_r_ = 0.1 ns, *t*_f_ = 0.1 ns, *p*_w_ = 4 ns, and *T*_per_ = 100 ns. Properly engineering the pulse amplitude, width, rise/fall times, and frequency induces the required transient temperature profile in the GST layer to enable reversible phase switching. The gold (Au) electrodes transport the electrical stimulus for Joule heating of the GST.

**Figure 9:**
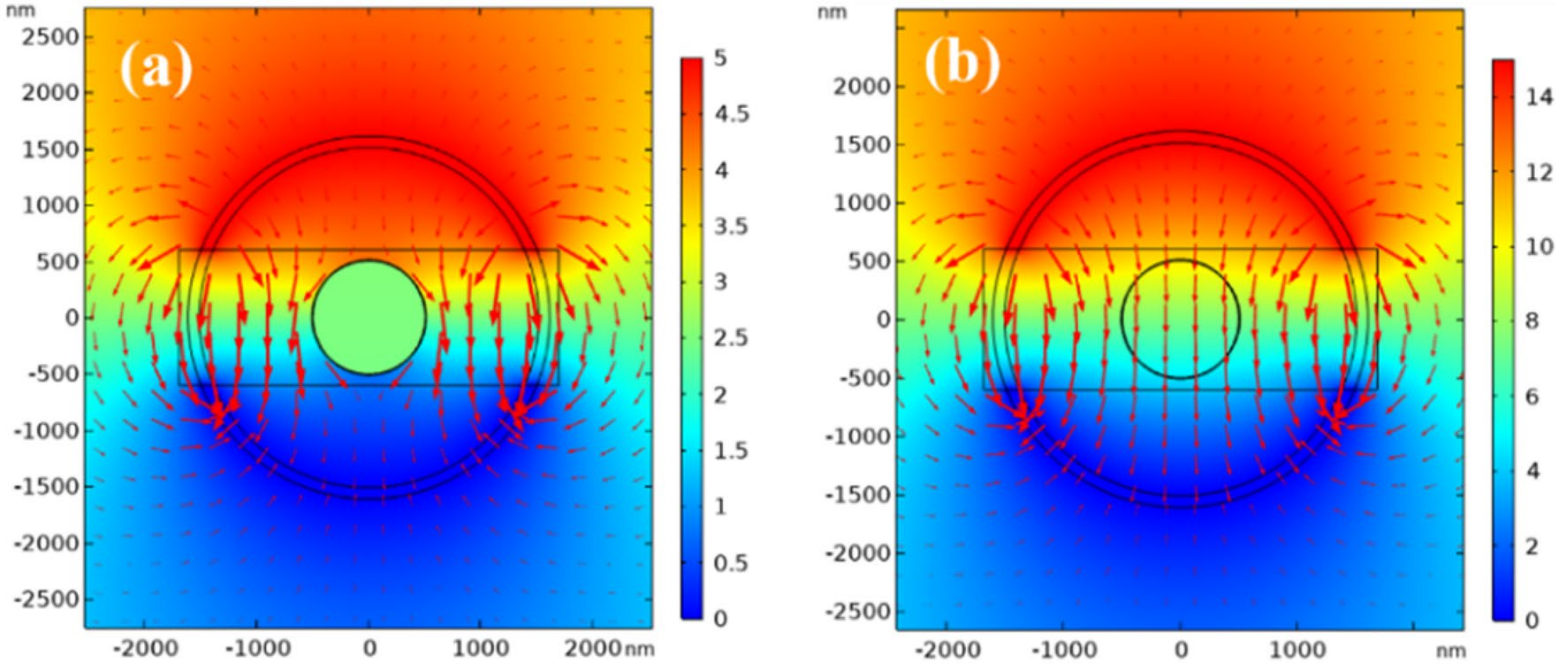
Electrical potential and current density (arrow) distributions in the cross-section of the optimized optical switch design during (**a**) amorphous-to-crystalline and (**b**) crystalline-to-amorphous phase transitions. The applied voltages are 5 V and 15 V, respectively.

The electrical conductivity of crystalline GST is nearly 1000 times higher than amorphous GST. Therefore, a higher voltage pulse is required to switch from the crystalline to amorphous phase. Figure 9 shows the simulated current density distribution between the Au electrode layers during phase change. In the amorphous-to-crystalline transition (Fig. 9a), a 5 V pulse produces high current density along the Au edges, with minimal current in the inner Si layer. However, for crystalline-to-amorphous switching at 15 V (Fig. 9b), uniform current density passes through both the GST and inner Si regions.

**Figure 10:**
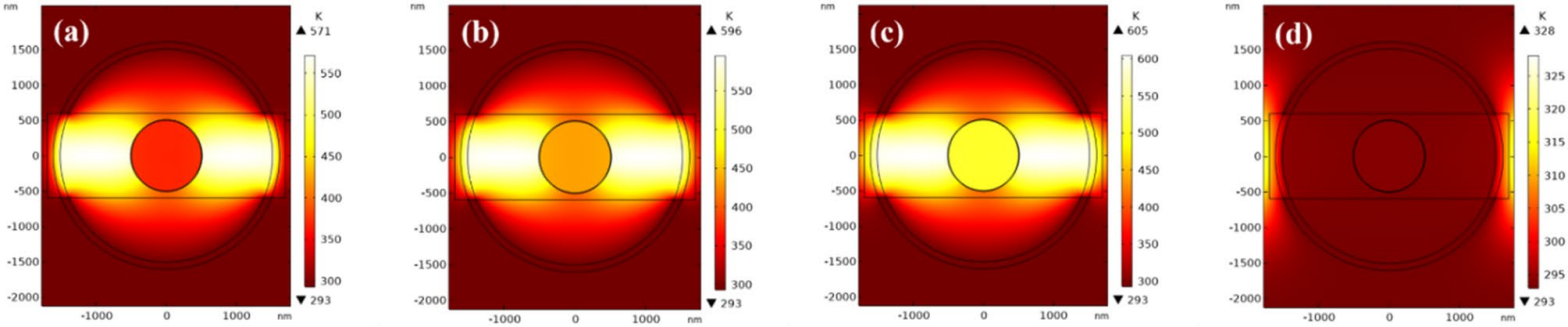
Temperature distribution in the cross-section of the optimized optical switch during amorphous-to-crystalline transition at times of (**a**) 25 ns, (**b**) 40 ns, (**c**) 95 ns, and (**d**) 270 ns.

Figure 10 shows simulated temperature distribution in the cross-section of the optimized optical switch during amorphous-to-crystalline phase change. The layer dimensions match the optimized optical design with thicknesses of 500 nm, 10 nm, 1000 nm, 100 nm, and 1200 nm for the inner Si, GST, outer Si, Au, and gap, respectively. A 5V pulse with 140 ns width and 160 ns cooling time was applied to induce crystallization. As seen in Fig. 10a, the GST layer reaches ~ 400 K after 25 ns, below the 413 K crystallization threshold. At 40 ns the temperature of GST surpasses the 413 K threshold temperature (Fig. 10b). At 95 ns (Fig. 10c), the GST is heated well above the threshold to sustain crystallization, which requires maintaining an elevated temperature sufficiently above the threshold temperature. After 140 ns, the voltage is turned off and the structure cools for 160 ns (Fig. 10d) to stabilize the crystalline phase.

**Figure 11:**
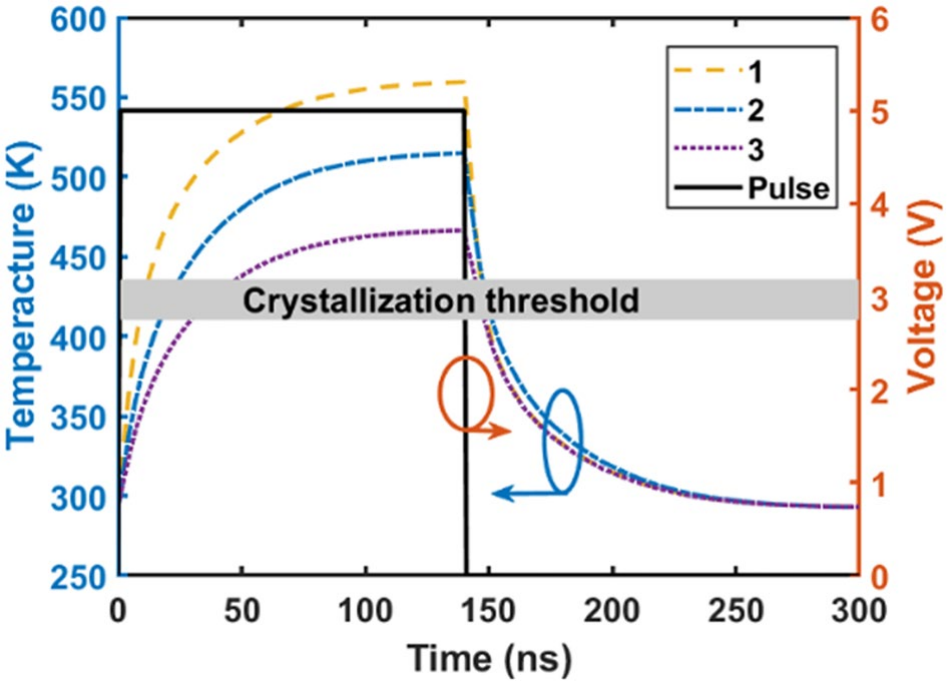
Applied voltage pulse and transient temperature profiles at points 1–3 (indicated in Fig. 1a) in the GST layer during crystallization. The thermal response exceeds the crystallization threshold for sufficient time to induce phase change.

Figure 11 shows the applied voltage pulse and resulting temperature curve over time at different locations in the GST layer during crystallization. A 5V, 140 ns pulse heats the structure above the 413 K crystallization threshold. Due to the gap in the Au layer, the temperature is non-uniform angularly. Three representative points were analyzed in each 90° quadrant, with the quadrants being equivalent by symmetry. Points 1–3 (see Fig. 1a) correspond to the maximum, average, and minimum temperatures in the GST layer. The minimum heating time above threshold should be ~ 4 ns for a 10 nm GST^49^. For times exceeding 100 ns, the temperatures at points 1–3 stabilize and remain above the threshold temperature. After turn-off the applied voltage, cooling returns the GST temperature to 293.15 K in 200 ns, which crystallize the GST layer.

**Figure 12:**
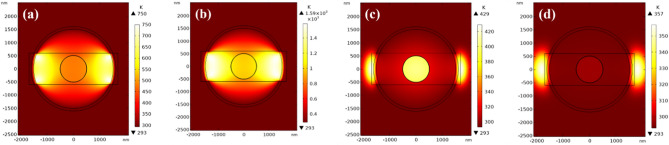
Temperature distribution in the cross-section of the optimized optical switch during crystalline-to-amorphous phase change at times of (**a**) 1 ns, (**b**) 4 ns, (**c**) 40 ns, and (**d**) 70 ns. The images reveal the transient thermal profile in the GST layer as it is heated above melting temperature and then cooled to stabilize the amorphous phase.

Figure 12 shows the simulated temperature distribution during crystalline-to-amorphous phase change. The transient response differs from crystallization due to the distinct phase switching behavior. Upon applying a 15V pulse, the temperature rapidly peaks within 4 ns and then gradually cools down. By 70 ns, the temperature in both the GST and Si materials drops below 300 K, allowing the completion of the amorphization process. During the amorphization process, the gap region plays a crucial role by concentrating the electric field, potentially raising the temperature of the adjacent silicon and gold layers above their melting points, which could adversely affect the optical properties. However, the optimized 1200 nm gap thickness mitigates this issue, ensuring that the silicon and gold remain in the solid state, preserving the desired optical characteristics.

**Figure 13:**
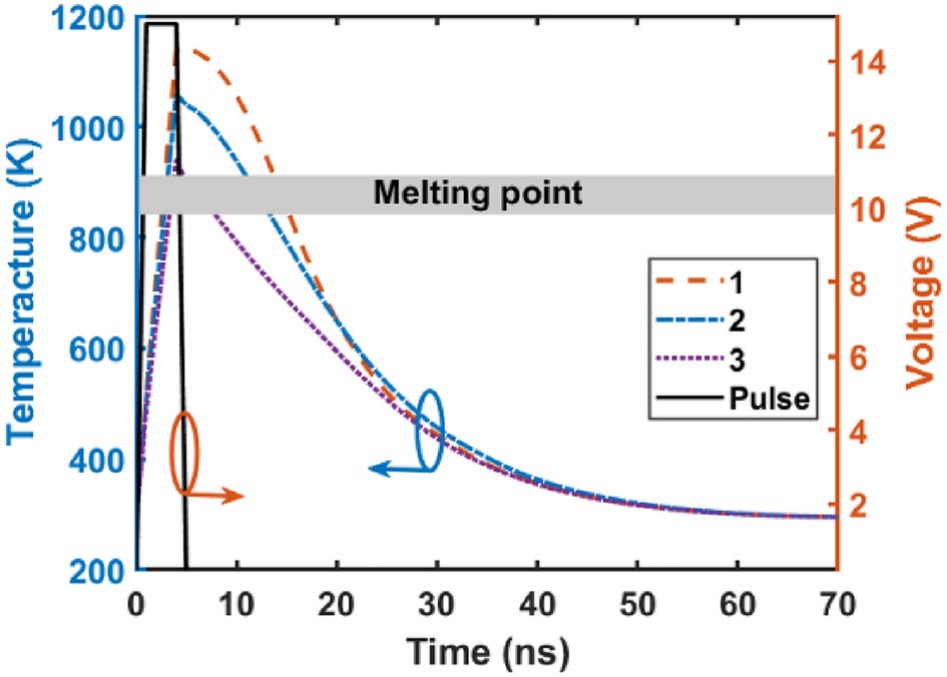
Applied voltage pulse and transient temperature profiles at points 1–3 (indicated in Fig. 1a) in the GST layer during crystalline-to-amorphous phase change. The thermal response exceeds the melting point of GST for sufficient time to induce amorphization.

Figure 13 illustrates the temperature response at different locations within the GST layer. The amorphization process requires heating the GST material above its melting point of approximately 819 K. However, for rapid amorphization on picosecond timescales, a higher applied voltage is necessary, as the atomic bonds within the material do not have sufficient time to rearrange at slower heating rates. Consequently, a 15 V, 4 ns pulse is applied, effectively heating the 10 nm GST layer above its melting point and enabling the amorphization process. Crucially, after 70 ns, all points within the GST layer cool below 300 K, facilitating the completion of the transition to the amorphous phase. This rapid cooling is essential to quench the disordered atomic structure and stabilize the material in the amorphous state, preventing undesired recrystallization.

Table 3 shows a comparison of simulation results for the proposed optical switch and some other modulators. The thermal simulation indicates that the optical switch has a bit rate of 3.5 MB/s at a wavelength of 1.55 μm. When the GST material switches between amorphous and crystalline phases, the maximum energy consumption is 30 nJ.

**Table 3:** Comparison of simulated performance metrics for the proposed optical switch and other phase change modulators based on GST at 1550 nm wavelength.

Device	IL (dB)	ER (dB)	BR	EC	V or I
GST modulator^53^	18	5.7	2.86 Mb/s	–	–
GST modulator^49^	0.94	5.4	–	194 pJ	1 & 5 V
GST modulator^54^	0.95	–	1 Mb/s	–	5 & 15 V
GST modulator^55^	1.23	12.6	1.88 Mb/s	8.6 nJ	–
Proposed optical switch	0.79	13.47	3.5 Mb/s	30 nJ	5 & 15 V

## Conclusion

This work introduces a cylindrical waveguide optical switch using GST as an active phase change material on a silicon photonics platform. The phase change dynamics were analytically modeled and simulated using FEM. The TE_01_ and fundamental HE_11_ modes were studied to enable low-loss propagation in the amorphous phase and high extinction in crystalline GST. Increasing the GST thickness improved optical absorption but also slowed down switching kinetics, reducing modulation speeds. With a 10 nm GST layer, competitive performance was achieved with 0.79 dB insertion loss (IL), 13.47 dB extinction ratio (ER), 30 nJ average power consumption (EC), and 3.5 Mb/s bit rate (BR). Compared to prior GST-based modulators, some parameters are improved. This comprehensive multiphysics analysis of an architecturally novel, cylindrically symmetric GST-silicon optical switch represents a significant advance towards reconfigurable nanophotonics devices. The results provide a foundation for future work using phase change materials in integrated photonic circuits and optical communications.

## Methods

The optical properties and performance of the proposed optical switch are analyzed using numerical techniques. 2D and 3D finite-element (FEM) and finite-difference time-domain (FDTD) methods are utilized to determine the modal and propagation characteristics, respectively. For FEM analysis of the modes, perfect electric conductor boundary conditions are applied at the exterior borders, with a tetrahedral mesh size of physics-controlled. The 3D FDTD simulation of light propagation employs perfectly matched layer (PML) outer boundaries and adaptive mesh refinement to minimize computation time and RAM requirements. Multiphysics electrical and thermal modeling utilizes 2D FEM, with thermally insulating boundaries and a triangular element mesh. Due to equation complexity, the analytical approach focuses only on the TE_01_ mode. By applying boundary conditions, the effective index is derived and the *H*_r_ field distribution is plotted. The analytical results are verified against FEM simulations, showing excellent agreement. Electrical current and thermal conduction physics are coupled for phase change simulations. The electrical and thermal properties of each layer determine the thermal profiles and amorphous-crystalline switching dynamics.

### Supplementary Information

Supplementary Information.

## Data Availability

The datasets used and/or analysed during the current study available from the corresponding author on reasonable request.
