# Supplementary material for: Multiphysics simulations of a cylindrical waveguide optical switch using phase change materials on silicon
Source: Sci Rep. 2024 May 10;14:10730. doi: 10.1038/s41598-024-61473-w (PMC11087545; doi:10.1038/s41598-024-61473-w)
Supplement: Supplementary file 1 — Supplementary Information. [file 41598_2024_61473_MOESM1_ESM.docx]

**Supplementary Information**

**Multiphysics Simulations of a Cylindrical Waveguide Optical Switch Using Phase Change Materials on Silicon**

**Alireza Malek Mohammad^1^, Mahmoud Nikoufard^1,2,*^, Senour Abdolghaderi^2^**

^1^Department of Electronics, Faculty of Electrical and Computer Engineering, Kashan, 8731753153, Iran

^2^Nanoscience and Nanotechnology Research Center, University of Kashan, Kashan, 8731753153, Iran

*  [mnik@kashanu.ac.ir](mailto:author@email.example)

**1. The elements of dispersion relation**

| $\left[ M \right]\left[ C \right]=\left[ 0 \right]\to\left[ \begin{matrix} a_{11} & a_{12} & 0 & 0 \\ a_{21} & a_{22} & a_{23} & 0 \\ a_{31} & a_{32} & a_{33} & a_{34} \\ a_{41} & a_{42} & a_{43} & a_{44} \end{matrix} \right]\left[ \begin{matrix} B_{21} \\ B_{22} \\ B_{32} \\ B_{42} \end{matrix} \right]=\left[ 0 \right]$ | (s1) |
| --- | --- |
| $\det\left( M \right)=a_{11}a_{22}a_{33}a_{44}-a_{11}a_{22}a_{34}a_{43}-a_{11}a_{23}a_{32}a_{44}$  ${+a}_{12}a_{21}a_{33}a_{44}+a_{12}a_{21}a_{34}a_{43}-a_{12}a_{23}a_{34}a_{41}+a_{11}a_{23}a_{34}a_{42}=0$ | (s2) |
| $a_{11}=\frac{J_{1}\left( \frac{U_{2}}{b}a \right)}{\frac{U_{2}}{b}}-\frac{J_{0}\left( \frac{U_{2}}{b}a \right)J_{1}\left( U_{1} \right)}{\frac{U_{2}}{b}J_{0}\left( U_{1} \right)}$ | (s2-a) |
| $a_{12}=\frac{Y_{1}\left( \frac{U_{2}}{b}a \right)}{\frac{U_{2}}{b}}-\frac{Y_{0}\left( \frac{U_{2}}{b}a \right)J_{1}\left( U_{1} \right)}{\frac{U_{1}}{a}J_{0}\left( U_{1} \right)}$ | (s2-b) |
| $a_{21}=\frac{J_{0}\left( U_{2} \right)J_{1}\left( \frac{U_{3}}{c}b \right)}{\frac{U_{3}}{c}J_{0}\left( \frac{U_{3}}{c}b \right)}-\frac{J_{1}\left( U_{2} \right)}{\frac{U_{2}}{b}}$ | (s2-c) |
| $a_{22}=\frac{J_{1}\left( \frac{U_{3}}{c}b \right)Y_{0}\left( U_{2} \right)}{\frac{U_{3}}{c}J_{0}\left( \frac{U_{3}}{c}b \right)}-\frac{Y_{1}\left( U_{2} \right)}{\frac{U_{2}}{b}}$ | (s2-d) |
| $a_{23}=\frac{Y_{1}\left( \frac{U_{3}}{c}b \right)\frac{U_{3}}{c}}{\frac{U_{3}}{c}J_{0}\left( \frac{U_{3}}{c}b \right)}-\frac{J_{1}\left( \frac{U_{3}}{c}b \right)Y_{0}\left( \frac{U_{3}}{c}b \right)\frac{U_{3}}{c}}{\frac{U_{2}}{b}J_{0}\left( \frac{U_{3}}{c}b \right)}$ | (s2-e) |
| $a_{31}=\frac{J_{0}\left( U_{2} \right)J_{1}\left( U_{3} \right)}{\frac{U_{3}}{c}J_{0}\left( \frac{U_{3}}{c}b \right)}-\frac{J_{1}\left( U_{2} \right)J_{0}\left( U_{3} \right)I_{0}\left( \frac{U_{4}}{d}c \right)}{\frac{U_{4}}{d}J_{0}\left( U_{3} \right)I_{0}\left( \frac{U_{4}}{d}c \right)}$ | (s2-f) |
| $a_{32}=-\frac{Y_{0}\left( U_{2} \right)J_{1}\left( U_{3} \right)}{\frac{U_{3}}{c}J_{0}\left( \frac{U_{3}}{c}b \right)}-\frac{Y_{0}\left( U_{2} \right)J_{0}\left( U_{3} \right)I_{1}\left( \frac{U_{4}}{d}c \right)}{\frac{U_{4}}{d}J_{0}\left( \frac{U_{3}}{c}b \right)I_{0}\left( \frac{U_{4}}{d}c \right)}$ | (s2-g) |
| $a_{33}=-\frac{Y_{1}\left( U_{3} \right)-Y_{0}\left( \frac{U_{3}}{c}b \right)J_{1}\left( U_{3} \right)}{J_{0}\left( \frac{U_{3}}{c}b \right)}-\frac{J_{0}\left( \frac{U_{3}}{c}b \right)Y_{0}\left( U_{3} \right){-Y}_{0}\left( \frac{U_{3}}{c}b \right)J_{1}\left( U_{3} \right)I_{1}\left( \frac{U_{4}}{d}c \right)}{\frac{U_{4}}{d}J_{0}\left( \frac{U_{3}}{c}b \right)I_{0}\left( \frac{U_{4}}{d}c \right)}$ | (s2-h) |
| $a_{34}=\frac{K_{1}\left( \frac{U_{4}}{d}c \right)+I_{1}\left( \frac{U_{4}}{d}c \right)K_{0}\left( \frac{U_{4}}{d}c \right)}{I_{0}\left( \frac{U_{4}}{d}c \right)}-K_{1}\left( U_{4} \right)Y_{0}\left( U_{3} \right)+K_{0}\left( \frac{U_{4}}{d}c \right)I_{1}\left( U_{4} \right)I_{0}\left( \frac{U_{4}}{d}c \right)$ | (s2-i) |
| $a_{41}=\frac{J_{0}\left( U_{2} \right)J_{0}\left( U_{3} \right)I_{1}\left( U_{4} \right)}{\frac{U_{4}}{d}J_{0}\left( \frac{U_{3}}{c}b \right)I_{0}\left( \frac{U_{4}}{d}c \right)}+\frac{J_{0}\left( U_{2} \right)J_{0}\left( U_{3} \right)I_{0}\left( U_{4} \right)K_{1}\left( U_{5} \right)}{\frac{U_{5}}{d}J_{0}\left( \frac{U_{3}}{c}b \right)I_{0}\left( \frac{U_{4}}{d}c \right)K_{0}\left( U_{5} \right)}$ | (s2-j) |
| $a_{42}=\frac{Y_{0}\left( U_{2} \right)J_{0}\left( U_{3} \right)I_{1}\left( U_{4} \right)}{\frac{U_{4}}{d}J_{0}\left( \frac{U_{3}}{c}b \right)I_{0}\left( \frac{U_{4}}{d}c \right)}+\frac{Y_{0}\left( U_{2} \right)J_{0}\left( U_{3} \right)I_{0}\left( U_{4} \right)K_{1}\left( U_{5} \right)}{\frac{U_{5}}{d}J_{0}\left( \frac{U_{3}}{c}b \right)I_{0}\left( \frac{U_{4}}{d}c \right)K_{0}\left( U_{5} \right)}$ | (s2-k) |
| $a_{43}=\frac{J_{0}\left( \frac{U_{3}}{c}b \right)Y_{0}\left( U_{3} \right)-Y_{0}\left( \frac{U_{3}}{c}b \right)J_{0}\left( U_{3} \right)I_{1}\left( U_{4} \right)}{\frac{U_{4}}{d}J_{0}\left( \frac{U_{3}}{c}b \right)I_{0}\left( \frac{U_{4}}{d}c \right)}+$  $\frac{J_{0}\left( \frac{U_{3}}{c}b \right)Y_{0}\left( U_{3} \right)-Y_{0}\left( \frac{U_{3}}{c}b \right)J_{0}\left( U_{3} \right)I_{0}\left( U_{4} \right)K_{0}\left( U_{5} \right)}{\frac{U_{5}}{d}J_{0}\left( \frac{U_{3}}{c}b \right)I_{0}\left( \frac{U_{4}}{d}c \right)K_{0}\left( U_{5} \right)}$ | (s2-l) |
| $a_{44}=\frac{K_{1}\left( U_{5} \right)J_{0}\left( \frac{U_{3}}{c}b \right)I_{0}\left( \frac{U_{4}}{d}c \right)K_{0}\left( U_{4} \right)-J_{0}\left( \frac{U_{3}}{c}b \right)K_{0}\left( \frac{U_{4}}{d}c \right)I_{0}\left( U_{4} \right)}{\frac{U_{5}}{d}J_{0}\left( \frac{U_{3}}{c}b \right)I_{0}\left( \frac{U_{4}}{d}c \right)K_{0}\left( U_{5} \right)}$ | (s2-m) |

**2. Thermal conductivity of silicon elements**

For silicon, the thermal conductivity, *k*, has a temperature dependence given by the following equation^1^.

| $k= k_{0}\left[ 1-B\left( \frac{T-T_{0}}{T} \right)^{A} \right]$ | (s3) |
| --- | --- |

where *k*_0_ is the thermal conductivity of silicon at room temperature *T*_0_, *T* is the temperature-dependent silicon temperature, and the constants *A* and *B* have values of 0.8705 and 1.093 respectively in the 300-1000 K range, and 0.42 and 0.93795 above 1000-1600 K. The melting point of silicon is 1686 K.

**3. Optimization of Au thickness and gap**

To determine the optimal Au layer thickness (*c* < *r* < *d*), simulations were performed by sweeping this parameter from 10 to 100 nm. The gap distance was fixed at 100 nm. Figure s1 shows the simulated real and imaginary parts of the effective refractive index (Re(*n*_eff_) and Im(*n*_eff_)) as a function of Au layer thickness for both amorphous and crystalline phases of the GST material. As shown in Fig. s1, varying the Au layer thickness has minimal effect on the real and imaginary parts of the effective refractive index (Re(*n*_eff_) and Im(*n*_eff_)) for both GST phases and modes. This indicates negligible optical power propagates in the Au layer, which is due to the optimization of the inner and outer Si layer dimensions. Based on these results and thermal simulations, the Au thickness is set to 100 nm. This enables voltage application without melting the device.

| **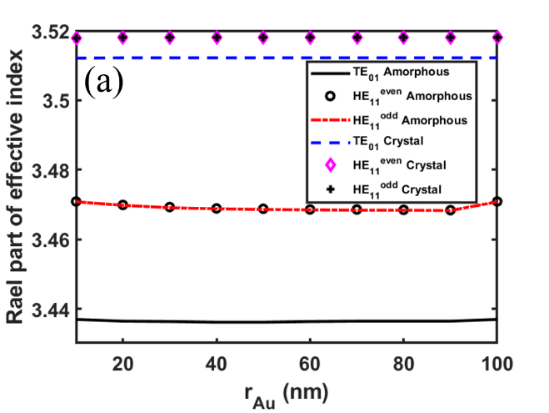** | **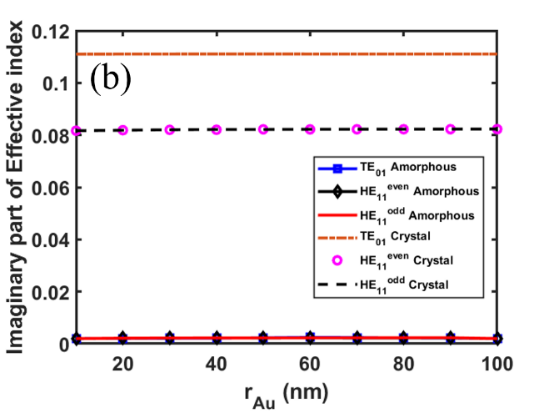** |
| --- | --- |
| **Figure s1.** Effective refractive index of the proposed optical switch as a function of Au layer thickness. **(a)** Real and **(b)** imaginary parts are shown for the HE_11_ and TE_01_ modes in both amorphous and crystalline phases of the GST. | |

To determine the optimal gap thickness, simulations were performed sweeping this dimension from 100 to 1000 nm. Figure s2 shows the resulting Re(*n*_eff_) and Im(*n*_eff_) for amorphous and crystalline GST phases. Increasing the gap thickness has a negligible effect on Re(*n*_eff_) and Im(*n*_eff_) for both GST phases, that remain essentially constant. This indicates the gap does not substantially alter the optical modes. The main role of the gap is to divide the Au layer to enable connecting electrodes for applying voltage. Since both Au and crystalline GST absorb optical power, the gap thickness is selected as 100 nm to minimize absorption while providing space for electrodes. This gap dimension enables efficient electrical switching while not perturbing the optical properties.

| 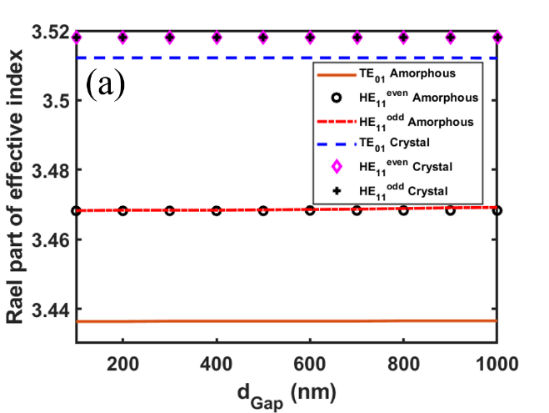 | 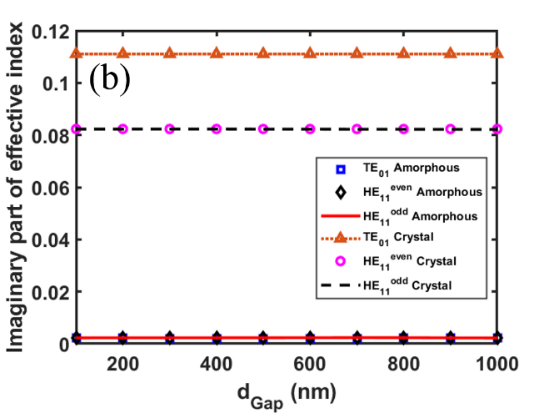 |
| --- | --- |
| **Figure s2.** Simulated effective refractive index of the proposed optical switch as a function of gap thickness. **(a)** Real and **(b)** imaginary parts are shown for the HE_11_ and TE_01_ modes in both amorphous and crystalline GST phases. | |

**3. Temperature properties of Si, Au, and GST materials**

As temperature variations induce phase change in the GST layer, the temperature-dependent properties of the Si, GST, and Au layers are listed in Tables s1-s3.

| **Table s1.** Temperature-dependent material properties for silicon layers. | | | |
| --- | --- | --- | --- |
| **Property** | **Variable** | **Value** | **Unit** |
| **Heat capacity at constant pressure** | *c* | ** | J/(kg·K) |
| **Density** | *ρ* | ** | kg/m³ |
| **Thermal conductivity** | *k* | * | W/(m·K) |
| **Electrical conductivity** | *σ* | 10000 [17] | S/m |
| **Coefficient of thermal expansion** | *α* | ** | 1/K |
| **Relative permittivity** | $\text{ϵ}_{\text{r}}$ | 12 [17] | 1 |
| A double asterisk (**) denotes material property data. A single asterisk (*) indicates experimental equations or measured data used as input parameters. | | | |

| **Table s2.** Temperature-dependent material properties for GST layer. | | | |
| --- | --- | --- | --- |
| **Amorphous Phase** | | **Crystalline Phase** | |
| **Property** | **Value** | **Property** | **Value** |
| **Electrical conductivity (**$\boldsymbol{\sigma}$**)** | 3 (S/m) | Electrical conductivity ($\sigma$) | 2770 (S/m) |
| **Thermal conductivity (k)** | 0.17 (W/(m.K)) | Thermal conductivity (k) | 0.5 (W/(m.K)) |
| **Density (**$\boldsymbol{\rho}$**)** | 5995 (kg/(m^3)) | Density ($\rho$) | 5995(kg/(m^3)) |
| **Relative permittivity (**$\boldsymbol{\epsilon}_{\boldsymbol{r}}$**)** | 16.0 | Relative permittivity ($\varepsilon_{r}$) | 33.3 |

For silicon, the thermal conductivity, *k*, has a temperature dependence^1^. Yu et al.^2^ investigated the relationship between GST thickness and required switching time above the crystallization threshold (413 K) and melting point (819 K). Thicker GST layers need longer dwell times above the crystallization temperature for phase change causing to decrease in the switching speed. Wang et al.^3^ studied the transient temperatures needed for amorphous-to-crystalline and crystalline-to-amorphous switching. Picosecond pulses can melt and quench crystalline GST into the amorphous phase since the atomic bonds do not have time to rearrange. However, slower nanosecond pulses are required for crystallization to allow resonant bonding. In summary, phase change dynamics depend strongly on GST thickness and heating/cooling rates ^2,4^. Thinner GST allows faster switching times. Proper thermal engineering is crucial for optimizing device performance.

| **Table s3.** Temperature-dependent material properties for Au layer. | | | |
| --- | --- | --- | --- |
| **Property** | **Variable** | **Value** | **Unit** |
| **Heat capacity at constant pressure** | *c* | - | J/(kg⋅K) |
| **Thermal conductivity** | *k* | - | W/(m⋅K) |
| **Electrical conductivity** | *σ* | - | S/m |
| **Relative permittivity** | $\text{ϵ}_{\text{r}}$ | -115.13+11.259i | 1 |

Experimental data were used to determine the temperature-dependent heat capacity, thermal conductivity, and electrical conductivity of gold ^2,5^. The heat capacity increases with temperature, while conductivity decreases linearly.

Applying an electrical pulse allows temporally controlling the phase change in GST. Table s4 shows the pulse characteristics for amorphous-to-crystalline and crystalline-to-amorphous switching. Here, *V*_src_ is the source voltage, *V*_off_ the offset voltage, *t*_d_ the delay time, *t*_r_ the rise time, *t*_f_ the fall time, *p*_w_ the pulse width, and *T*_per_ the period. Proper engineering of the pulse amplitude, width, rise/fall time, and frequency enables achieving the required temperature profile in the GST to induce reversible phase switching. The Au electrodes transport the electrical stimulus.

**4. Pulse parameters applied to the electrodes**

| **Table s4.** Utilized electrical pulse parameters for amorphous-to-crystalline and crystalline-to-amorphous phase switching of GST material. | | | | |
| --- | --- | --- | --- | --- |
| **Amorphous to Crystalline Phase** | | 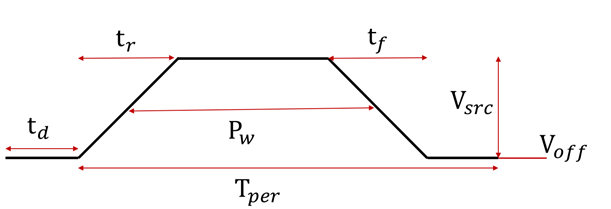 | **Crystalline to Amorphous Phase** | |
| *V*_src_ (V) | 5 |  | *V*_src_ (V) | 15 |
| *V*_off_ (V) | 0 |  | *V*_off_ (V) | 0 |
| *t*_d_ (ns) | 0 |  | *t*_d_ (ns) | 0 |
| *t*_r_ (ns) | 1 |  | *t*_r_ (ns) | 0.1 |
| *t*_f_ (ns) | 1 |  | *t*_f_ (ns) | 0.1 |
| *P*_w_ (ns) | 140 |  | *P*_w_ (ns) | 4 |
| *T*_per_ (ns) | 300 |  | *T*_per_ (ns) | 100 |

**References**

1. Prakash, C., Thermal conductivity variation of silicon with temperature. *Microelectronics Reliability*. **18(4)**, 333 (1978).

2. Yu, Z. et al., Ultracompact electro-optical modulator-based Ge_2_Sb_2_Te_5_ on silicon. *IEEE Photonics Technology Letters*, **30(3)**, 250-253 (2017).

3. Wang, W. J., et al. Fast phase transitions induced by picosecond electrical pulses on phase change memory cells. *Applied Physics Letters*, **93(4)** (2008).

4. Takahashi, Y. et al., Heat capacity of gold from 80 to 1000 K. *Thermochimica acta*, **109(1)**, 105-109 (1986).

5. Zaki, A. O. K. et al. Hybrid plasmonic electro-optical modulator. *Applied Physics A,* **122(4)**, 473 (2016).
